# Dermatophytosis caused by *Microsporum gypseum* in infants: report of four cases and review of the literature*

**DOI:** 10.1590/abd1806-4841.20165044

**Published:** 2016

**Authors:** Beatriz da Silva Souza, Débora Sarzi Sartori, Carin de Andrade, Edna Weisheimer, Ana Elisa Kiszewski

**Affiliations:** 1 Universidade Federal de Ciências da Saúde de Porto Alegre (UFCSPA) – Porto Alegre (RS), Brazil; 2 Private Clinic – Pelotas (RS), Brazil; 3 Private Clinic – Joinville (SC), Brazil; 4 Private Clinic – Estrela (RS), Brazil

**Keywords:** Infant, Microsporum, Tinea

## Abstract

Dermatophytosis caused by *Microsporum gypseum* is rare, especially in infants, with few published cases. Diagnosis in this age group is frequently delayed. We review the literature and report 4 new cases of tinea of glabrous skin caused by M. gypseum mimicking eczema in infants. Considering new and previously reported cases, half of patients were exposed to sand, emphasizing the importance of this transmission vehicle in this age group. In conclusion, although rare, dermatophytosis by M. gypseum should be part of the differential diagnosis of inflammatory dermatosis in infants. A clinical suspicion and the availability of culture are keys to the diagnosis.

## INTRODUCTION

*Microsporum gypseum* is a geophilic fungus that has a worldwide distribution and rarely causes disease in humans. This fungus may be found in dogs and cats (which can be asymptomatic carriers), in sick human beings, and especially in contaminated soil.^1-4^ Dermatophytosis caused by *M. gypseum* usually manifests as an inflammatory mycosis that typically affects the glabrous skin and scalp, especially in children.^5^ Rarely, it can present as onychomycosis.^4^ Only few cases of dermatophytosis caused by *M. gypseum* in children under two years have been published in the literature.^2-9^ The aim of this study was to verify, taking into account a review of the literature and a new series of cases, if the dermatophytosis caused by *M. gypseum* present preferred topography in this age group, if the sand is important as a source of infection for infants and if there is a tendency to late diagnosis in our midst.

## CASE REPORT

**Figure 1:**
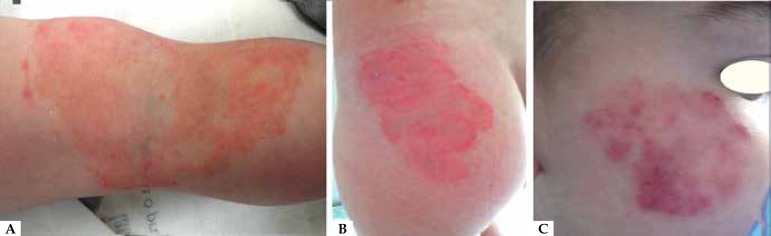
(**A**) Case 1 - male patient with well-defined erythematous-desquamative plaques in popliteal fossa with few pustules; (**B**) Case 3 - female patient with erythematous-desquamative plaques with polycyclic edges and pustules on the buttocks; (**C**) Case 4 - male patient with erythematous-desquamative plaques with irregular edges and pustules in malar region

The cases were treated between April 2009 and May 2013. We assessed three males and one female patients, aged between 15 and 24 months. All were from the metropolitan region of Porto Alegre, Rio Grande do Sul, Brazil. Two patients had contact with sand. All patients presented development of dermatosis for more than three months and all exhibited erythematous scaly plate with pustules (Table 1 and Figure 1).

**Table 1:** Infants with dermatophytosis by *Microsporum gypseum*

Case	Age	Sex	Lesion site	Time of evolution	Gateway and possible source of infection	KOH examination	Culture
1	15 months	M	popliteal fossa	5 months	Contact with sand	Negative	*M. gypseum *
2	24 months	M	knee	1 month	Excoriation	Negative	*M. gypseum *
3	20 months	F	gluteus	6 months	Contact with sand	Negative	*M. gypseum *
4	20 months	M	face	4 months	Unknown	Septate hyphae of fungi	*M. gypseum *

**Figure 2:**
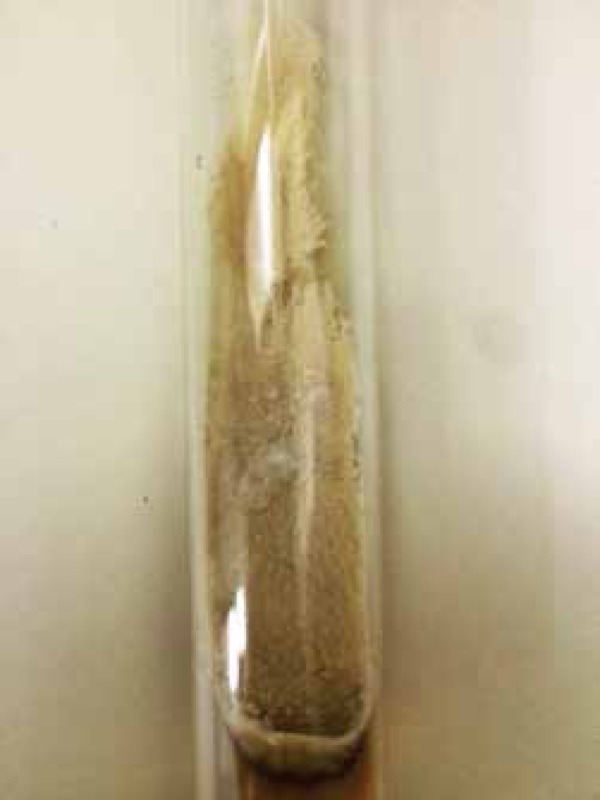
Powdery filamentous colony, sand-colored and with "sugar with cinnamon" aspect

Three of four patients (patients 1, 2 and 4) received the wrong diagnosis of infected atopic dermatitis, and one patient (patient 3), of diaper dermatitis. All were mistakenly treated with topical steroids prescribed by the pediatrician. Scales and pustule contents were collected for direct mycological examination (DME). Microscopy was performed after clarification with KOH solution 20%. Septated ant branched hyphae were identified in only one patient. All patients had positive culture for *M. gypseum*. Macroscopic examination of the culture showed powdery filamentous colony, sand-colored and with "sugar with cinnamon" aspect. Microscopic inspection revealed abundant multiseptated macroconidia, symmetrical, ellipsoidal and with rounded ends (Figure 2). Three patients received oral treatment with terbinafine (62.5 mg per day) for seven days. All patients were treated with topical imidazole (three with bifonazole and one with clotrimazole) for 30 to 60 days.

## DISCUSSION

Dermatophytoses are common fungal infections in tropical countries and may represent a public health problem. The dermatophytosis caused by *M. gypseum* represents from 0.7% to 5% of all dermatophytosis.^4^ The infection outbreaks are exceptional and have been reported in Italy, Colombia, Brazil and Spain.^4,5,7,10^ When this occurs, dermatophytosis caused by *M. gypseum* can be assigned to a specific virulence of the fungus, to a high concentration of the fungus in the soil, to a low resistance of the host, or to the presence of previous skin lesion.^2,7^ Infants may be especially vulnerable, because in addition to play often on the floor, they belong to an age group with increased susceptibility to infections. Moreover, the creation of pets is a common practice, which causes contamination of the floor and increases the risk of infection.^2,4^ Only eight cases involving infants were published in English and Portuguese literature (Table 2).^2-9^

**Table 2:** Dermatophytosis review by *Microsporum gypseum* in infants

Author/country	N. of cases	Age	Sex	Lesion site	Evolution	Gateway or possible source of infection	KOH examination	Culture
Haga R, 2002/Japan	1	24 months	M	Scalp	*	Small cut	Negative	M. gypseum
Metkar A, 2010/India	1	25 days	M	Trunk, thighs and legs	23 days	*	Hyaline hyphae	M. gypseum
Romano C, 2009/Italy	1	18 months	M	Knee	*	Excoriation	*	M. gypseum
Severo LC, 1989/Brazil	2	Case 1:12 months; case 2: 24 months	M M	Face Gluteus	* *	Sand Sand	Septate hyphae of fungi	M. gypseum
Criado PR, 2005/Brazil	1	14 months	M	Nose	2 months	Contact with dog	Septate hyphae of fungi	M. gypseum
KamalamA, 1981/India	1	2 days	F	Face	15 days	Soil	Mycelium and arthrospores	M. gypseum
Romano C, 2000/Italy	1	24 months	F	Ear	3 months	Soil	Hyphae (dermatophytes)	M. gypseum

The intense history of contact with sand was present in 50% of our cases, data coinciding with those described in the literature.^5,8,9^ In Brazil, it is common for parents to take their children to play in sandboxes in playgrounds. It is also common for infants to play in sandboxes in preschool. A frequent problem is that most of the sandboxes are not protected to prevent the entry of domestic animals such as dogs and cats. It is likely that these factors, combined with other environmental factors and the increased susceptibility of infants to infection, put our country as a risk for this dermatophytosis. The cases reported here, added to the ones previously published, show that more than 50% of them occurred in Brazil. In addition, our patients had lesions located predominantly in the lower limbs and gluteal region, reinforcing the importance of sand in the transmission of this fungus. In the literature, the most affected sites were the lower limbs and head.^1,4,6-8^

Lesions caused by *M. gypseum* are generally characterized by erythematous scaly plates with pustules inside or on the edges. So, they can easily simulate different inflammatory dermatitis and secondary infection.^2,6^ The prescription of topical corticosteroids and antibiotics is common and often delay diagnosis.^3^ Other clinical patterns can occur, such as erythematous plaque with well-defined edges, characteristic of mycosis, or atypical lesions, such as plates with sclerodermiform, eczematid, lichenoid, psoriasiform or crusted aspects.^1,4^ Among infants, the differential diagnosis should be made mainly with seborrheic eczema, atopic eczema and bacterial infection.^1,7^

The diagnosis is made with DME and culture for fungi. The treatment may be carried out with topical imidazole or terbinafine (for at least four weeks). The association of oral antifungals can be indicated in large or symptomatic cases (due to the inflammatory process) in order to speed up the resolution of the condition. In this case, terbinafine, itraconazole, fluconazole, or griseofulvin may be used.^3,4,6,9^

We report the cases of four infants with dermatophytosis caused by *M. gypseum*, with a mean time of evolution before diagnosis of 3.5 months, showing a clear delay in diagnosis. This delay can occur: (1) because the inflammatory nature of the lesions can mimic other inflammatory dermatoses; (2) because this is an age in which dermatophytosis are infrequent; or (3) because DME can be negative. In summary, this paper warns dermatologists of the need to include this differential diagnosis in the evaluation of inflammatory skin lesions in infants, especially if there is a history of exposure to sand or soil. Finally, the clinical suspicion and the availability of culture are essential for diagnosis.

## References

[1] Lopes JO, Alves SH, Benevenga JP (1992). Dermatofitose humana por Microsporum gypseum no interior do Rio
Grande do Sul estudo clínico. An Bras Dermatol.

[2] Haga R, Suzuki H (2002). Tinea capitis due to Microsporum gypseum. Eur J Dermatol.

[3] Metkar A, Joshi A, Vishalakshi V, Miskeen AK, Torsekar RG (2010). Extensive neonatal dermatophytoses. Pediatr Dermatol.

[4] Romano C, Massai L, Gallo A, Fimiani M (2009). Microsporum gypseum infection in the Siena area in
2005-2006. Mycoses.

[5] Severo LC, Conci LMA, Amaral AA (1989). Microsporum gypseum- Relato de surto de infecção e
isolamento do solo. An Bras Dermatol.

[6] Criado PR, Costa AR, Vasconcellos C, Oliveira Ramos R, Silva CS, Souza SF (2005). Tinea faciei in an infant caused by Microsporum gypseum
simulating a dry impetigo. Pediatr Dermatol.

[7] García-Martos P, Ruiz-Aragón J, García-Agudo L, Linares M (2004). Dermatophytoses due to Microsporum gypseum report of eight cases
and literature review. Rev Iberoam Micol.

[8] Kamalam A, Thambiah AS (1981). Tinea facei caused by Microsporum gypseum in a two days old
infant. Mykosen.

[9] Romano C, Asta F, Massai L (2000). Tinea incognito due to Microsporum gypseum in three
children. Pediatr Dermatol.

[10] Sierra de Arroyave B, Yepes A, Arenas J, Santamaría de Uribe L, Restrepo A (1977). Epidemic outbreak of tinea corporis due to Microsporum
gypseum. Mycopathologia.

